# Near-complete genome sequences of multiple genotype 1 African swine fever virus isolates from 2016 to 2018 in Cameroon

**DOI:** 10.1128/mra.00978-23

**Published:** 2024-03-13

**Authors:** Edward Spinard, Abel Wade, Hermann Unger, Nenkam Robert, Francis J. Mayega, Vattipally B. Sreenu, Ana Da Silva Filpe, Daniel Mair, Manuel V. Borca, Douglas P. Gladue, Charles Masembe

**Affiliations:** 1 U.S. Department of Agriculture, Agricultural Research Service, Foreign Animal Disease Research Unit, Plum Island Animal Disease Center, Orient, New York, USA; 2 U.S. Department of Agriculture, Agricultural Research Service, Foreign Animal Disease Research Unit, National Bio and Agro-Defense Facility, Unit Name, Manhattan, Kansas, USA; 3 National Veterinary Laboratory (LANAVET), Garoua, Cameroon; 4 Animal Production and Health Section, Joint FAO/IAEA Division for Nuclear Applications in Food and Agriculture, Department of Nuclear Sciences and Applications, International Atomic Energy Agency, Vienna International Centre, Vienna, Austria; 5 Laboratoire National Veterinaire (LANAVET), Garoua, Cameroon; 6 College of Natural Sciences, Makerere University, Kampala, Uganda; 7 MRC-University of Glasgow Centre for Virus Research, Glasgow, United Kingdom; Katholieke Universiteit Leuven, Leuven, Belgium

**Keywords:** ASFV, ASF, Cameroon, African swine fever, African swine fever virus

## Abstract

African swine fever virus has been endemic in Cameroon since 1982. Here, we announce the sequences of Cameroon/2016/C1, Cameroon/2016/C5, Cameroon/2017/C-A2, Cameroon/2018/C02, and Cameroon/2018/CF3, five genotype 1 African swine fever virus genomes collected from domestic pigs between 2016 and 2018.

## ANNOUNCEMENT

African swine fever virus (ASFV) of the genus *Asfivirus* and Asfarviridae family has been endemic in Cameroon since its first outbreak of African swine fever in 1982 (1). Historically, ASFV has been classified into 24 genotypes based on the partial sequencing of the B646L (p72) gene (2). Derivatives of the current pandemic strain (ASFV Georgia 2007/01) belong to genotype 2 and have been circulating in Asia (3), Europe (4), and the Dominican Republic (5). Yet, as of early 2020, only ASFV isolates belonging to genotype 1 have been observed in Cameroon (6, 7). However, in 2020, Nigeria, which shares a border to the west of Cameroon, reported an outbreak caused by a unique genotype 2 strain, highlighting the need for continued ASFV surveillance programs in the area (5, 8–10).

ASFV isolates were passed once in primary swine macrophage cultures produced from blood, as previously described (11). Viral DNA was extracted from infected macrophage cultures using the MagMax Pathogen RNA/DNA Kit (Applied Biosystems) as previously described (12). For sequencing, CpG-methylated (host) DNA was depleted using a NEBNext Microbiome Enrichment Kit E2612L (New England Biolabs, MA, USA), followed by library construction using a KAPA LTP Library Preparation Kit 796188001 (Roche Diagnostics, IN, USA) and sequencing on a NextSeq 500 instrument (Illumina, CA, USA) (13). Sequence analysis was performed using CLC Genomics Workbench v23 software (CLCBio, Waltham, MA, USA). Reads were trimmed for ambiguous nucleotides (max = 2), quality (Quality limit = 0.05), minimum length (50 nt), and nucleotide composition (20 and 5 nts removed from the 5′ and 3′ ends, respectively) resulting in 744,540 (Cameroon/2016 /C1), 1,234,348 (Cameroon/2016 /C5), 1,588,982 (Cameroon/2017 /C-A2), 49,740,870 (Cameroon/2018 /C02), and 28,694,650 (Cameroon/2018/CF3) as described in Table 1; paired-end reads ranging in size from 50 to 126 nt were assembled using the default parameters of CLC Genomic Workbench’s *de novo* assembly and resulted in one scaffold per isolate. *N*s were inserted in scaffold regions and represent an approximate size gap. Reads were mapped back to the scaffolds, and unmapped regions on the 5′ and 3′ ends of the genome were trimmed. From the read map, the consensus sequence was extracted resulting in a genome length of 183,210 (Cameroon/2016 /C1), 183,381 (Cameroon/2016 /C5), 183,395 (Cameroon/2017 /C-A2), 183,390 (Cameroon/2018 /C02), and 183,406 (Cameroon/2018/CF3) nucleotides with a 13×, 10×, 81×, 13×, and 189 × coverage, respectively. All genomes had a GC content of 38.5%. Whole-genome alignments, performed using CLC Genomics Workbench, revealed that the genomes were 99.93% (Cameroon/2017 /C-A2 vs Cameroon/2016 /C1) to 99.99% (Cameroon/2018 /C-F3 vs Cameroon/2016 /C-A2) similar. BlastN against the NCBI database revealed Benin 97/1 (NC_044956.1) to be the closest match, ranging from 99.51% (Cameroon/2016 /C5) to 99.56% (Cameroon/2016 /C1) similarity based on whole-genome nucleotide alignment. Annotations were transferred from Benin 97/1 using the Genome Annotation Transfer Utility (14) using the default parameters resulting in 152 (Cameroon/2016 /C1), 151 (Cameroon/2016 /C5), 155 (Cameroon/2017 /C-A2), 151 (Cameroon/2018 /C02), and 155 (Cameroon/2018/CF3) predicted genes (15).

**TABLE 1:** ASFV isolates sequenced and collection dates and locations in Cameroon

GenBank accession number	Sample	Collection date	Location	Genotype	Closest RefSeq match	Sequencing reads
OQ971722	Cameroon/2018/C-F3	2018	Cameroon: Lokoundj	1	Benin 97/1 (NC_044956.1)	SRR25629145
OQ971723	Cameroon/2016/C1	2016	Cameroon: Yaound III	1	Benin 97/1 (NC_044956.1)	SRR25629144
OQ971724	Cameroon/2016/C5	2016	Cameroon: Mfou	1	Benin 97/1 (NC_044956.1)	SRR25629142
OQ971725	Cameroon/2017/C-A2	2017	Cameroon: Mezam	1	Benin 97/1 (NC_044956.1)	SRR25629141
OQ971726	Cameroon/2018/C02	2018	Cameroon: Bertoua I	1	Benin 97/1 (NC_044956.1)	SRR25629143

## Data Availability

The genome sequences for isolates Cameroon/2018/CF3, Cameroon/2016 /C1, Cameroon/2016 /C5, Cameroon/2017 /C-A2, and Cameroon/2018 /C02 have been deposited in NCBI GenBank under the accession no. OQ971722.1, OQ971723.1, OQ971724.1, OQ971725.1, and OQ971726.1, respectively. Raw sequence data can be found in the GenBank SRA under BioProject accession no. PRJNA1005237.
